# Hepatic stellate cells promote immunotolerance following orthotopic liver transplantation in rats via induction of T cell apoptosis and regulation of Th2/Th3-like cell cytokine production

**DOI:** 10.3892/etm.2012.801

**Published:** 2012-11-06

**Authors:** ZHIJUN JIANG, YING CHEN, XIAONIN FENG, JIANWEN JIANG, TIANXIANG CHEN, HAIYANG XIE, LIN ZHOU, SHUSEN ZHENG

**Affiliations:** Division of Hepatobiliary and Pancreatic Surgery, Department of Surgery, First Affiliated Hospital, School of Medicine, Zhejiang University, Hangzhou 310003, P.R. China

**Keywords:** hepatic stellate cells, liver transplantation, Fas/FasL, apoptosis, immunotolerance

## Abstract

Hepatic stellate cells (HSCs) have been demonstrated to have immunoinhibitory activity. The aim of this study was to investigate the role of HSCs in the development of immunotolerance following liver transplantation. A rat liver transplantation tolerance model [donor Lewis into recipient Dark Agouti (DA)] and rejection (donor DA into recipient Lewis) was established. On the 7th day following transplantation, the HSCs and T cells were isolated from the rats of either the tolerance or rejection group and cultured together. The apoptosis rate of the T cells was determined 24 h later by flow cytometry following staining with anti-CD3 mAb and Annexin V-FITC/PI. Additionally, the FasL expression of HSCs was determined by flow cytometry following staining with anti-FasL mAb. The protein levels of IL-2, TNF-α, TGF-β and IL-10 in the supernatant collected from mixed lymphocyte reaction cultures of HSCs and T cells for 5 days were measured using ELISA assays. HSCs isolated from the tolerance group had a higher T-cell apoptosis induction activity compared with those of the rejection group. The activity of the HSCs was partially reversed by FasL blocking mAb. Accordingly, the FasL expression level of HSCs in the tolerance group was revealed to be higher than that of the rejection group. Moreover, HSCs stimulated IL-10 and TGF-β production in the tolerance group. This study suggests that HSCs are involved in liver transplantation immune tolerance via the induction of T-cell apoptosis partially mediated by the Fas/FasL pathway and the activation of Th2/Th3-like cell cytokine production.

## Introduction

Although organ transplantation has been successful for decades, graft rejection and immunosuppression (IS)-derived chronic toxicity remain as key problems which need to be overcome. However, in comparison with the majority of transplant recipients who have extreme difficulty achieving an IS-free state following transplantation, a significantly higher proportion of liver transplant recipients achieve clinical operational tolerance (1). Moreover, liver allografts are able to protect co-transplanted organs from rejection (2–4), suggesting that the liver, unlike other solid organs such as the kidney or heart, is an immunoprivileged organ. Notably, although liver allografts are the most spontaneously accepted transplantations in a number of species, hepatocyte allografts alone are acutely rejected (5,6), suggesting that liver non-parenchymal cells (NPCs), including resident dendritic cells (DCs), liver sinusoid endothelial cells (LSECs), Kupffer cells (KCs) and hepatic stellate cells (HSCs), are considerably involved in liver immunotolerance. Previous studies have indicated that NPCs mediate immunosuppresion via a variety of mechanisms, including the secretion of anti-inflammatory cytokines and the induction of T-cell apoptosis (7), However, the underlying mechanism is not yet completely understood.

HSCs (vitamin A-storing cells, lipocytes, interstitial cells, fat-storing cells and Ito cells), exist in the space between the hepatocytes and LSECs of the hepatic lobule and are well known for their functions of regulating retinoid homeostasis and participating in the pathogenesis of liver fibrogenesis. In addition, HSCs have been demonstrated to be antigen-presenting cells (APCs) and tolerogenic (8). Activated HSCs express negative co-stimulator B7-H1 which inhibits T-cell responses via the mediation of T-cell apoptosis (9). Previous studies have revealed that co-transplanted HSCs protect islet allografts from rejection and attenuate the severity of graft-versus-host disease (10,11), suggesting that the HSCs have immunosuppressive properties. The present study revealed that HSCs are involved in liver transplant immunotolerance via T-cell apoptosis partly mediated by the Fas/FasL pathway and the regulation of TGF-β and IL-10 production.

## Materials and methods

### Animals

Male Dark Agouti (DA) and Lewis rats, aged between 10 and 12 weeks and weighing 220–250 g (Vital River Laboratory Animal Technology Co., Ltd., Beijing, China) were maintained in a pathogen-free animal facility. The rats were allowed free access to tap water and food. The animal procedures were approved by the Institutional Animal Care Committee.

### Orthotopic liver transplantation

Previous studies have shown that liver grafts are spontaneously accepted in Lewis to DA transplantations, while DA to Lewis liver allograft recipients suffer from severe rejection (12). The present study included a tolerance group (Lewis into DA, n=5) and rejection group (DA into Lewis, n=5). The liver transplantation was performed according to the ‘2 cuff technique’ of Kamada and Calne (13). Briefly, after anesthesia and systemic heparinization, the livers were removed from the donors and prepared in 4°C Ringer’s solution. The grafts were then implanted into the recipients by anastomosis of the suprahepatic vena cava using a continuous everting suture, reconstruction of the portal vein and infra-hepatic vena cava by the cuff technique and connection of bile duct by an end-to-end anastomosis over an indwelling stent without hepatic artery reconstruction. After surgery, the recipients were kept warm by lighting and had free access to food and water. Rats deaths within 5 days after transplantation were considered to be due to technical failures and hence excluded from the study.

### Histological examination

Tissue specimens from the liver grafts were fixed in 4% formalin, embedded in paraffin and used for histological examination. The specimens were then sliced into 5-*μ*m sections and stained with hematoxylin and eosin (H&E) for routine histological examination.

### Isolation of HSCs

On the 7th day after transplantation, the recipient rats were sacrificed. A 0.5×1.0 cm liver tissue specimen was obtained from each liver graft for histological examination. HSCs were then extracted from the left liver tissue as previously described (14–16). Briefly, the grafts underwent serial *in situ* perfusions with 70 ml 0.1% pronase at a flow rate of 10–15 ml/min for 7 min and 60 ml 0.05% collagenase IV (Sigma, St. Louis, MO, USA) at a flow rate of 10–15 ml/min for 20 min. The liver tissue was then digested in 50 ml buffer solution containing collagenase IV, pronase and DNase (Sigma), followed by density gradient centrifugation and 11% Nycodenz (Axis-Shield PoC, Oslo, Norway) gradient centrifugation. The harvested HSCs were resuspended in high glucose Dulbecco’s modified Eagle’s medium (DMEM; Gibco-BRL, Grand Island, NY, USA) containing 20% fetal calf serum (FCS). The viability of HSCs was >90% as determined using trypan blue exclusion and the purity of HSCs ranged from 90 to 95% as determined by desmin immunostaining. The typical light microscopic appearance of a lipid droplet was as described previously (17). The HSCs were cultured in DMEM containing 20% FCS for 7 days for further study.

### Preparation of T cells

The spleens of the recipient rats were removed on the 7th day after transplantation and single spleen cell (SC) suspensions were prepared. Following lysis of red blood cells and density gradient centrifugation, T cells were isolated and purified using an adherence culture in DMEM containing 10% FCS and a nylon wool column.

### FasL expression in HSCs

HSCs (2.5×10^4^) were incubated with mitomycin C (10 *μ*g/ml) for 30 min at 37°C in a 5% CO_2_-humidified air atmosphere. Then, HSCs were stained with Hamster monoclonal anti-rat FasL-specific IgG (eBioscience, San Diego, CA, USA) followed by a second fluorescein-labeled antibody (BD Pharmingen, San Diego, CA, USA). The FasL expression levels of the HSCs were determined by flow cytometry according to the manufacturer’s instructions.

### Flow cytometry analysis for alloreactive T-cell apoptosis

After incubating with mitomycin C (10 *μ*g/ml) for 30 min, the HSCs (2.5×10^4^) were resuspended in 100 *μ*l DMEM containing 10% FCS and then co-cultured with 100 *μ*l T cells (5×10^5^) in the presence or absence of FasL blocking mAb for 24 h. All cultures were incubated at 37°C in a 5% CO_2_-humidified air atmosphere. The nonadherent cells were isolated and the apoptotic T cells stained with anti-CD3 mAb (eBioscience) and Annexin V-FITC/PI (BD Pharmingen) were determined by flow cytometry according to the manufacturer’s instructions.

### Cytokine quantitation

A two-way mixed lymphocyte reaction (MLR) was performed and 2.5×10^5^ Lewis and 2.5×10^5^ DA SCs were co-cultured in 96-well plates (200 *μ*l) in DMEM containing 10% FCS, 100 U/ml penicillin and 100 mg/ml streptomycin, in the presence of 2.5×10^4^ HSCs. This co-culture was incubated at 37°C in a 5% CO_2_-humidified air atmosphere for 5 days. The IL-2, IL-10, TNF-α and TGF-β levels in the supernatant of this co-culture were quantified using the respective ELISA kits (Biosource International Inc, Camarillo, CA, USA). A standard curve using recombinant cytokine was generated for each assay.

### Statistical analysis

The data are presented as the mean ± standard deviation (SD). The statistical significance of the parametric data was determined using the Student’s t-test. P<0.05 was considered to indicate a statistically significant difference.

## Results

### Histological characteristics of the liver grafts

**Figure 1:**
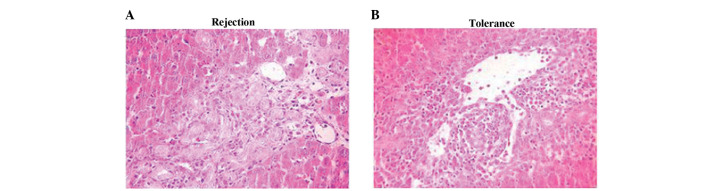
Histological characteristics of the liver grafts on the 7th postoperative day. (A) H&E staining of the rejection group: T-cell infiltration of the portal tracts, bile duct damage, vacuolar degeneration, karyopycnosis and necrosis of hepatocytes were observed. (B) H&E staining of the tolerance group: minimal T-cell infiltration without the involvement of the portal tracts was observed and hepatic parenchyma exhibited no significant damage (×400). H&E, hematoxylin and eosin.

Histological analysis confirmed that the liver grafts of the rejection group underwent serious acute rejection, while those of the tolerance group did not. In the rejection group, the H&E staining results showed severe acute rejection, which was characterized by extensive T-cell infiltration of the portal tracts; bile duct damage; vacuolar degeneration, karyopycnosis and even necrosis of hepatocytes. Meanwhile, the tolerance group exhibited only minimal T-cell infiltration without portal tract involvement and the hepatic parenchyma exhibited no significant damage (Fig. 1).

### HSCs induce T-cell apoptosis through the Fas/FasL pathway

**Figure 2:**
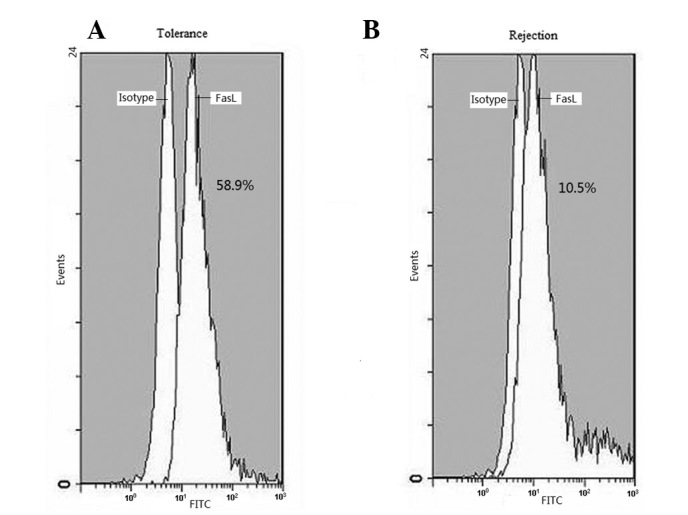
Flow cytometry analysis showing FasL expression in HSCs. FasL expression in HSCs from (A) the tolerance group was higher than in (B) the rejection group. HSCs, hepatic stellate cells.

**Figure 3:**
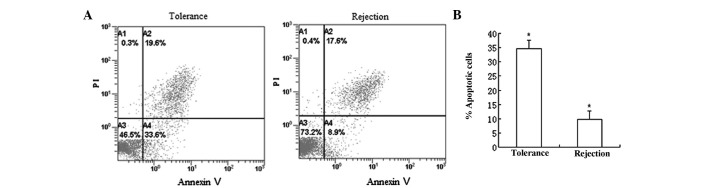
Flow cytometry analysis of the apoptosis rate of T cells after co-culturing with HSCs for 24 h. (A) Representative flow cytometry analysis of apoptotic T cells which were respectively co-cultured with HSCs from the tolerance group and rejection group. (B) Frequencies of apoptotic T cells after co-culturing with HSCs from the tolerance group and from the rejection group were analysed. Results are expressed as mean ± SD and are representative of five independent experiments. T cells co-cultured with HSCs from the tolerance group had a higher apoptosis rate than those co-cultured with HSCs from the rejection group. ^*^P<0.05. HSCs, hepatic stellate cells.

**Figure 4:**
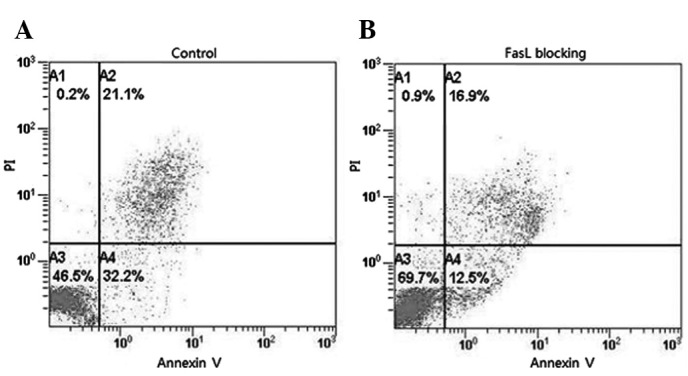
FasL blocking mAb partially but significantly inhibited T-cell apoptosis in the tolerance group. (A) T cells and HSCs were co-cultured without FasL blocking mAb. (B) T cells and HSCs were co-cultured with the FasL blocking mAb. HSCs, hepatic stellate cells.

**Figure 5:**
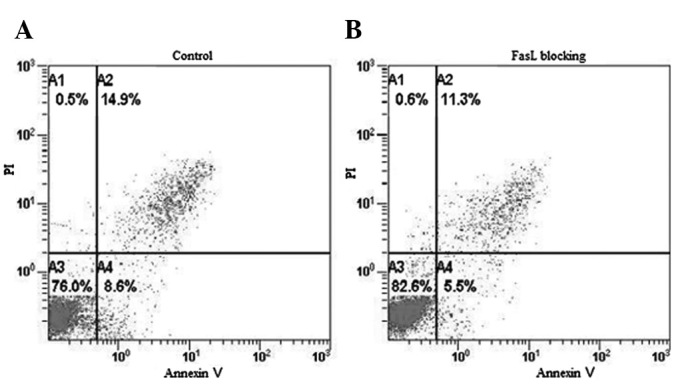
FasL blocking mAb did not notably reduce T-cell apoptosis in the rejection group. (A) T cells and HSCs were co-cultured without FasL blocking mAb. (B) T cells and HSCs were co-cultured with FasL blocking mAb. HSCs, hepatic stellate cells.

As T-cell apoptosis is a well-established mechanism of liver immunotolerance (18), we hypothesized that HSCs may contribute to liver immunotolerance by inducing T-cell apoptosis. The results indicated that the HSCs of the tolerance group had significantly higher FasL expression than the rejection group (Fig. 2). Furthermore, the results also showed that the HSCs of the tolerance group were able to markedly enhance the apoptosis of T cells, suggesting that the immunosuppressive effect of HSCs may induce T-cell apoptosis (Fig. 3). The Fas/FasL is a well-known pathway of apoptosis (19). To determine whether Fas/FasL was critical in HSC-induced T-cell apoptosis, HSCs were co-cultured with T cells in the presence or absence of FasL blocking mAb. FasL blocking mAb partially but significantly inhibited T-cell apoptosis in the tolerance group (P<0.05) and had little effect in the rejection group (P>0.05; Figs. 4 and 5).

### HSCs promote Th2/Th3-like cell cytokine production in the tolerance group

**Figure 6:**
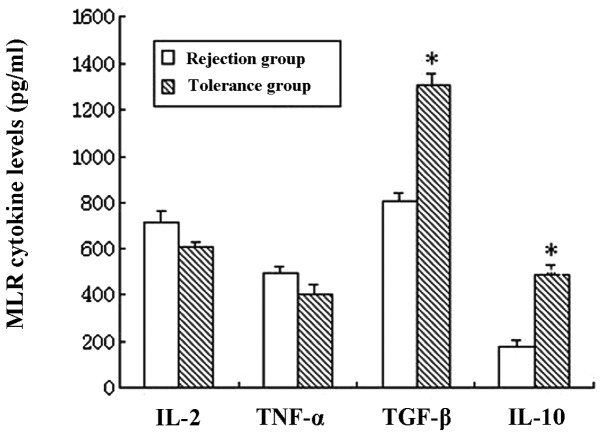
IL-2, TNF-α, TGF-β and IL-10 cytokine levels in the supernatant of MLR cultures in the presence of HSCs and T cells were measured by ELISA assays. Results are presented as mean ± SD and are representative of five independent experiments. TGF-β and IL-10 levels were notably higher in the tolerance group (^*^P<0.05). There were no significant differences of IL-2 and TNF-α levels between the two groups (P>0.05). MLR, mixed lymphocyte reaction; HSCs, hepatic stellate cells.

The IL-2, TNF-α, TGF-β and IL-10 cytokine levels in the supernatant of the MLR cultures in the presence of HSCs were measured using ELISA assays. The results indicated that there were no significant differences in the Th1 cytokine (IL-2 and TNF-α) levels between the tolerance and rejection groups (P>0.05). However, in the tolerance group, the Th2-like cell cytokine IL-10 and Th3-like cell cytokine TGF-β levels were markedly increased (P<0.05; Fig. 6).

## Discussion

Unlike other solid organs, liver allografts may be spontaneously accepted without IS in a number of species, including humans, demonstrating that the liver is an immunoprivileged organ (1,20–22). Moreover, an accumulating amount of evidence suggests that NPCs, such as DCs, KCs and LSECs, are critical in liver transplant immunotolerance (23–25). HSCs are a type of NPC, well known for their key role in liver fibrogenesis. Additionally, HSCs have been demonstrated to be immunoregulating (8–10).

In the present study, the activated HSCs from the tolerance group had an increased T-cell apoptosis-inducing activity, indicating that activated HSCs have an immune suppressive function, since induced T-cell apoptosis is a significant mechanism in the development of immune tolerance (18). The present data also showed that in the tolerance group, the expression of FasL by HSCs was significantly higher than in the rejection group. Moreover, FasL blocking mAb partially but significantly reversed HSC-induced T-cell apoptosis in the tolerance group but not in the rejection group, suggesting that the Fas/FasL pathway is associated with HSC-induced T-cell apoptosis. It is generally accepted that Fas and FasL interactions are key to cell apoptosis and maintain the immunoprivilege (19). Moreover, other studies have revealed that the Fas system is involved in liver transplant immune regulation. Specifically, FasL expressed by infiltrating cells induces liver cell apoptosis during acute rejection following liver transplantation. However, the increased expression of FasL in liver allografts results in immunotolerance by combining with Fas expressed by the infiltrating lymphocytes (26,27). This phenomenon may be explained by the expression of FasL gradually switching from infiltrating cells to hepatocytes. Sun *et al* observed that KCs in the liver downregulate the T-cell response via the Fas/FasL pathway following liver transplantation. The authors results indicated that the Fas/FasL pathway was involved in the immunotolerance of liver transplantation (24). However, FasL may not be the only molecule involved in the immunosuppressive effect of HSCs, since the blocking of FasL only partially reversed HSC-induced T-cell apoptosis. Studies have further demonstrated that the upregulation of B7-H1 suppresses T-cell proliferation, promotes T-cell apoptosis and induces the production of various cytokines by combining with programmed death-1 (PD-1) B and T lymphocyte attenuator. Therefore, B7-H1 is considerably involved in peripheral immune tolerance and tumor immune evasion (28,29). B7-H1 negatively regulates the immune system and inhibits T-cell activity, mainly at the effect phase since B7-H1 receptor PD-1 is inducibly expressed on activated T cells (30–32). Deficiencies of B7-H1 lead to the accumulation of CD8^+^ T cells in the liver, suggesting a role for B7-H1 in the regulation of T-cell homeostasis (33). A study by Yu *et al* showed that quiescent HSCs express very low levels of B7-H1, while B7-H1 expression in HSCs may be notably increased by various stimuli and the inhibition of B7-H1 may partially reduce HSC-induced T-cell apoptosis (9). Therefore, we propose that the B7-H1 and Fas/FasL pathways are involved in HSC-induced immune suppression.

The present study also showed that there were higher IL-10 and TGF-β levels in the supernatant of the MLR cultures of HSC and T cells from the tolerance group. We hypothesize that HSCs may drive the T-cell subset differentiation of Th2/Th3 cells or activate the production of inhibitory cytokines, such as IL-10 and/or TGF-β, by HSCs. Th1-like cell cytokines mediate cellular immunity and enhance rejection, while Th2-like cell cytokines downregulate the activity of Th1-like cells and cytotoxic T lymphocytes (CTL) and attenuate post-transplantation rejection. It has been reported that IL-10 and TGF-β contributed to liver transplant immunotolerance (34,35). Studies have also demonstrated that KCs and LSECs negatively regulate the immune response by secreting TGF-β (35). Therefore, HSCs may regulate the immune response following liver transplantation by regulating Th2/Th3-like cell cytokine production.

In conclusion, the present study revealed that HSCs contribute to liver transplant immunotolerance by inducing T-cell apoptosis and stimulating Th2/Th3-like cell cytokine production. This immunosuppressive activity of HSCs provides a supplementary mechanism for the development of immunotolerance following liver transplantation.
